# Antcin K Inhibits TNF-α, IL-1β and IL-8 Expression in Synovial Fibroblasts and Ameliorates Cartilage Degradation: Implications for the Treatment of Rheumatoid Arthritis

**DOI:** 10.3389/fimmu.2021.790925

**Published:** 2021-12-15

**Authors:** David Achudhan, Shan-Chi Liu, Yen-You Lin, Chien-Chung Huang, Chun-Hao Tsai, Chih-Yuan Ko, I-Ping Chiang, Yueh-Hsiung Kuo, Chih-Hsin Tang

**Affiliations:** ^1^ Graduate Institute of Biomedical Science, College of Medicine, China Medical University, Taichung, Taiwan; ^2^ Department of Medical Education and Research, China Medical University Beigang Hospital, Yunlin, Taiwan; ^3^ Department of Pharmacology, School of Medicine, China Medical University, Taichung, Taiwan; ^4^ Division of Immunology and Rheumatology, Department of Internal Medicine, China Medical University Hospital, Taichung, Taiwan; ^5^ Department of Sports Medicine, College of Health Care, China Medical University, Taichung, Taiwan; ^6^ Department of Orthopedic Surgery, China Medical University Hospital, Taichung, Taiwan; ^7^ Department of Pathology, China Medical University Hospital, Taichung, Taiwan; ^8^ Department of Chinese Pharmaceutical Sciences and Chinese Medicine Resources, China Medical University, Taichung, Taiwan; ^9^ Department of Biotechnology, Asia University, Taichung, Taiwan; ^10^ Chinese Medicine Research Center, China Medical University, Taichung, Taiwan

**Keywords:** rheumatoid arthritis, tumor necrosis factor alpha, interleukin 1 beta, interleukin 8, antcin K, collagen-induced arthritis, rheumatoid arthritis synovial fibroblast, *Antrodia cinnamomea*

## Abstract

Extracts from Taiwan’s traditional medicinal mushroom, *Antrodia cinnamomea*, exhibit anti-inflammatory activities in cellular and preclinical studies. However, this paper is the first to report that Antcin K, a triterpenoid isolated from *A. cinnamomea*, inhibits proinflammatory cytokine production in human rheumatoid synovial fibroblasts (RASFs), which are major players in rheumatoid arthritis (RA) disease. In our analysis of the mechanism of action, Antcin K inhibited the expression of three cytokines (tumor necrosis factor alpha [TNF-α], interleukin 1 beta [IL-1β] and IL-8) in human RASFs; cytokines that are crucial to RA synovial inflammation. Notably, incubation of RASFs with Antcin K reduced the phosphorylation of the focal adhesion kinase (FAK), phosphoinositide 3-kinase (PI3K), protein kinase B (AKT) and nuclear factor-κB (NF-κB) signaling cascades, all of which promote cytokine production in RA. Intraperitoneal injections of Antcin K (10 mg/kg or 30 mg/kg) attenuated paw swelling, cartilage degradation and bone erosion, and decreased serum levels of TNF-α, IL-1β, IL-8 in collagen-induced arthritis (CIA) mice; in further experiments, IL-6 levels were similarly reduced. The inhibitory effects of Antcin K upon TNF-α, IL-1β and IL-8 expression in human RASFs was achieved through the downregulation of the FAK, PI3K, AKT and NF-κB signaling cascades. Our data support clinical investigations using Antcin K in RA disease.

## Introduction

Rheumatoid arthritis (RA) is an autoimmune and chronic inflammatory disease with a complex pathogenesis that is not fully understood, but is known to involve the infiltration of inflammatory cells into the joints and subsequent swelling, synoviocyte proliferation, cartilage damage and bone erosion (1, 2). The production of proinflammatory cytokines from activated RA synovial fibroblasts (RASFs) disrupts the microenvironment that mediates bone homeostasis (3, 4). RASFs are considered to be the main catabolic factor in cartilage bone degradation, as they stimulate the production of proinflammatory cytokines typically associated with RA disease, including tumor necrosis factor alpha (TNF-α), interleukin 1 beta (IL-1β), IL-8 and IL-6 (5).

Symptomatic pain and inflammation associated with RA disease is generally prescribed nonsteroidal anti-inflammatory drugs (NSAIDs) and steroidal anti-inflammatories (corticoids) in addition to existing RA treatment, while synthetic or biologic disease-modifying antirheumatic drugs (DMARDs) in combination with corticoids can be used as first-line treatments in newly-diagnosed RA to achieve disease remission (6). NSAIDs and corticoids are commonly used in RA management. However, their long-term safety is yet to be confirmed, as a recent Cochrane Review of randomized clinical trial evidence on the efficacy and safety of these anti-inflammatories in the long-term treatment of RA concluded that the quality of the NSAID evidence was very low overall and meta-analysis evidence was lacking for corticoids (6). Importantly, long-term pharmacotherapy in RA disease is often associated with significant side effects, such as cytopenia, poor tolerability, rash, and occasionally liver damage occurring with traditional DMARDs (7), while data from observational studies indicate higher risks of cardiovascular disease, infections, diabetes mellitus and mortality with glucocorticoids (8), and side effects relating to biologic treatment with tumor necrosis factor (TNF) inhibitors can include severe infection, sepsis, tuberculosis, lymphoma or demyelinating disorders (9).

Novel therapeutic alternatives with good tolerability are needed to halt the progression of RA disease (10). Traditional Chinese medicinal plants contain functional compounds that can cure various disease conditions (11) and are an important source of drug development. In particular, the traditional Chinese medicine (TCM) remedy *Lianhua Qingwen* markedly downregulates messenger RNA (mRNA) expression of cytokines TNF-α and IL-6 and of the chemokines monocyte chemoattractant protein-1 [(MCP-1), also referred to as chemokine (CC-motif) ligand 2 (CCL2)] and C-X-C motif chemokine ligand 10 [(CXCL10), also known as interferon gamma-induced protein 10 (IP-10)] in human hepatocellular carcinoma (Huh-7) cells infected with severe acute respiratory syndrome coronavirus 2 (SARS-CoV-2) (12).

*Antrodia cinnamomea* is a species of mushroom endemic to Taiwan that has been used for hundreds of years in TCM and *A. cinnamomea* extracts are well known for their hepatoprotective, antioxidative, antihypertensive, antihyperlipidemic, immunomodulatory, anticancer and anti-inflammatory activities (13–15). In this study, we demonstrate that a triterpenoid isolated from *A. cinnamomea*, Antcin K, exhibits anti-inflammatory effects in cellular and preclinical experiments investigating RA disease. Interestingly, we found that Antcin K downregulated the production of proinflammatory cytokines including TNF-α, IL-1β and IL-8 in RASFs *via* the FAK, PI3K, AKT and NF-κB signaling cascades. Furthermore, Antcin K ameliorated cartilage degradation in mice with collagen-induced arthritis (CIA). Our report is the first to explain how Antcin K inhibits cartilage degradation *in vitro* and also the production of proinflammatory cytokines *in vivo*. According to our evidence, Antcin K appears to have therapeutic potential in RA disease.

## Materials and Methods

### Materials

Antibodies against TNF-α (A11534), IL-1β (SC-7884), IL-8 (ab18672), p-FAK (Thr202/Tyr204; SC-7383), FAK (SC-5298), p-85 (SC-1637), p-AKT (Thr308; SC-16646-R), AKT (SC-5298), p-p65 (Ser536; SC-101752), p-65 (SC-8008), and β-actin (SC-58673) were purchased from Santa Cruz (Santa Cruz, CA, USA). Activators Angiotensin II (a FAK activator), 740-YP (a PI3K activator), SC-79 (an AKT activator) and prostratin (an NF-κB activator) were all purchased from Santa Cruz (Santa Cruz, CA, USA). Cell culture supplements were purchased from Invitrogen (Carlsbad, CA, USA). A Human Inflammation Antibody Array kit was supplied by RayBiotech, Inc. Human TNF-α, IL-1β and IL-8 ELISA kits were purchased from R&D Systems (MN, USA).

### Cell Culture

Immortalized RASFs (MH7A cells) were bought from the Riken Cell Bank (Ibaraki, Japan) and cultured as a single culture in RPMI-1640 medium supplemented with 10% fetal bovine serum (FBS) and penicillin/streptomycin 100 U/mL. Cell incubation was performed in a humidified atmosphere of 37°C, 5% CO_2_. The cells were passaged when they had grown to 80% confluence.

### MTT Assay

RASFs were seeded at a density of 5 × 10^3^ cells/well into 96-well plates and incubated with Antcin K (0, 0.3, 1, 3, or 10 μM) for 24 h, then incubated in 3-(4,5-dimethylthiazol-2-yl)-2,5-diphenyltetrazolium bromide (MTT) solution for 2 h. Dimethyl sulfoxide (DMSO) was added to the solution and absorbance at 550 nm was measured with a microplate reader (BioTek, Winooski, VT, USA).

### Quantitative Real-Time PCR for mRNA

After incubating the RASFs with Antcin K (0, 0.3, 1, 3, or 10 μM) for 24 h, total RNA was extracted from the RASFs using TRIzol™ reagent. The qPCR analysis was performed as per an established protocol (16–18). RNA concentration was measured using a NanoVue Plus™ Spectrophotometer (Biochrom Ltd., Cambridge, UK). 1 μg of total RNA was reverse-transcribed to complementary DNA (cDNA), which was then synthesized by the MMLV reverse transcription system (Invitrogen, Carlsbad, CA, USA) and mixed with Fast SYBR^®^ Green Mix. Gene expression was examined by the StepOnePlus™ Real-Time PCR System. GAPDH served as the internal control and the primers used in the qPCR assays are listed in **Table 1**.

**Table 1:** Sequences of RT-PCR primers.

GAPDH	Forward	AAT GGACAACTGGTCGTGGAC
	Reverse	CCCTCCAGGGGATCTGTTTG
TNF-α	Forward	CCTCTCTCTAATCAGCCCTCTG
	Reverse	GAGGACCTGGGAGTAGATGAG
IL-1β	Forward	ATGATGGCTTATTACAGTGGCAA
	Reverse	GTCGGAGATTCGTAGCTGGA
IL-8	Forward	CTGCGCCAACACAGAAATTATTGTA
	Reverse	CTGCGCCAACACAGAAATTATTGTA

### Western Blot Analysis

The RASFs were incubated with Antcin K (0, 0.3, 1, 3, or 10 μM) for 24 h, then washed with PBS and protein was extracted using RIPA buffer containing protease inhibitors. The Western blot analysis was performed as per an established protocol (19–22). The total proteins were quantified by the Thermo Scientific™ Pierce™ BCA Protein Assay Kit. Cell lysates were separated by SDS-PAGE electrophoresis then transferred to polyvinylidene difluoride (PVDF) membranes. The blots were blocked with 4% BSA, then incubated with primary antibodies TNF-α, IL-1β, IL-8, p-FAK, FAK, p-85, p-AKT, AKT, p-p65, p-65 and β-actin overnight before being subjected to three consecutive washes in a mixture of tris-buffered saline and polysorbate 20 (TBST) and then the blots were incubated with horseradish peroxidase (HRP) conjugated secondary antibodies at room temperature for 1 h. Enhanced chemiluminescent imaging of the blots was visualized with the UVP Biospectrum system (UVP, Upland, CA, USA).

### Enzyme-Linked Immunosorbent Assay (ELISA)

The RASFs were plated in 6-well dishes and grown to confluence. The culture medium was then exchanged with serum-free RPMI medium. Cells were pretreated for 30 min with pharmacological activators Angiotensin II (a FAK activator), 740-YP (a PI3K activator), SC-79 (an AKT activator) and prostratin (an NF-κB activator) to stimulate the RSAFs, before 24 h of Antcin K (0, 0.3, 1, 3, or 10 μM) treatment. The conditioned medium was collected and stored at –20°C until use. Secreted expression of TNF-α, IL-1β and IL-8 was examined using ELISA kits for TNF-α, IL-1β and IL-8 (R&D Systems, MN, USA), according to the manufacturer’s protocols.

Serum levels of TNF-α, IL-1β, IL-8 and IL-6 in CIA mice were examined using ELISA kits for TNF-α and IL-1β (R&D Systems, MN, USA), IL-8 (MyBioSource, Inc, San Diego, USA) and IL-6 (Max™ Set Deluxe Kits, BioLegend, USA) according to the manufacturers’ protocols.

### CIA Mouse Model

Forty C57BL/6J mice (aged 8-10 weeks) were obtained from the National Laboratory Animal Centre (Taipei, Taiwan) and randomly allocated to the four study groups (n=10 mice in each group). CIA was induced according to our previous report (23, 24), and the study protocol was approved by the Institutional Animal Care and Use Committee of China Medical University. After receiving two immunizations, 95% of the CIA group developed severe arthritis. Intraperitoneal injections of Antcin K (10 or 30 mg/kg; see the study timeline in (**Figure 7A**) were administered on alternative days for a total of 4 weeks. Arthritis severity was assessed in each knee by plethysmometer measurements. The mice were sacrificed after 42 days of treatment. The phalanges and ankle joints were removed and fixed in 4% paraformaldehyde for micro-computed tomography (micro-CT) analysis.

### Human Synovial Tissue and Synovial Fluid Samples

Human RA synovial tissues and synovial fluid samples (N=6) was obtained from patients with RA during knee replacement surgery and non-arthritic synovial tissue was obtained at arthroscopy after trauma joint derangement. The study protocol was approved by the Institutional Review Board of China Medical University Hospital (CMUH108-REC3-039). All patients completed written informed consent before study enrollment.

### Histopathological Analysis

Histopathological changes were examined by hematoxylin and eosin (H&E) and Safranin-O staining using a light microscope. For immunohistochemical (IHC) staining, 5-µm sections were prepared from paraffin-embedded tissue, deparaffinized in xylene and rehydrated with ethanol or citrate buffer (pH 6.0) for 20 min at 95°C. The specimens were stained with specific primary antibodies anti-TNF-α (A11534), anti-IL-1β (SC-7884), or anti-IL-8 (ab18672). All specimens were separately scored by two researchers in a blinded manner and the inter-rater reliability was assessed. **Supplementary Table 1** presents the results from microscopic evaluation of inflammation scores and gives a detailed analysis of pathology findings in ankle joint synovium and adjacent tissue from healthy control mice and CIA mice (untreated or treated with Antcin K) in slides stained with H&E, according to previous research (25, 26). All of the slides were independently evaluated by two pathologists blinded to treatment.

### Statistical Analysis

All statistical analyses were carried out using GraphPad Prism 5.0 (GraphPad Software) All results are represented as the mean ± standard deviation (SD) of at least six independent experiments. The Student’s *t*-test compared the means between experimental groups. The statistical difference was considered to be significant if the *p*-value was < 0.05.

## Results

### Upregulation of Proinflammatory Cytokines in Human RA Tissue and Plasma

**Figure 1:**
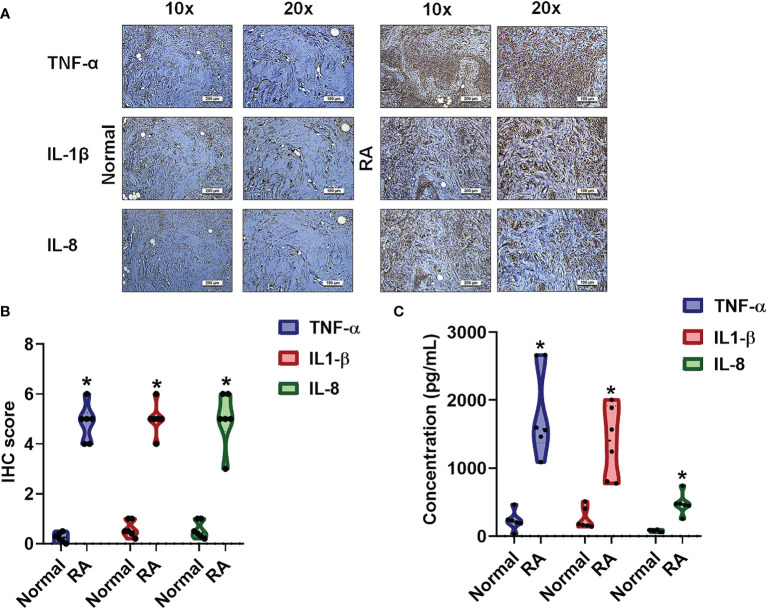
The identification of cytokines that interfere with RA severity. **(A, B)** RA and normal synovial tissue specimens were analyzed by IHC. **(C)** Cytokine plasma levels were upregulated in RA patients. ELISA assessments of blood samples (N=6) from RA patients and healthy volunteers determined levels of TNF-α, IL-1β and IL-8. Results are expressed as the mean ± SD. *P < 0.05 compared with controls.

In RA, proinflammatory cytokines are the principal pathological factors underlying the destruction of bone architecture and tissue damage at the site of inflammation (27). We observed aggressive proinflammatory cytokine activities in RA synovial tissue and fluid samples compared with normal healthy samples. IHC staining revealed higher levels of TNF-α, IL-1β and IL-8 in human RA tissue compared with samples from healthy controls (**Figures 1A, B**). Similarly, ELISA assay data revealed significantly higher expression of these proinflammatory cytokines in human RA plasma compared with control plasma (**Figure 1C**).

### Antcin K Lowers the Production of Proinflammatory Cytokines in RASFs

**Figure 2:**
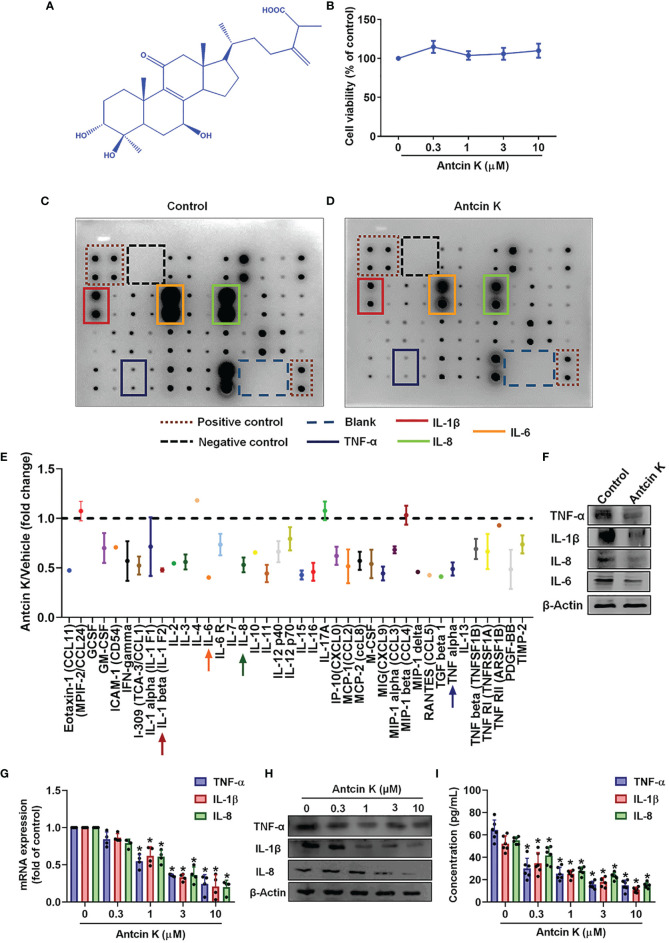
Antcin K inhibits TNF-α, IL-1β and IL-8 expression in RASFs. **(A)** Chemical structure of compound Antcin K. **(B)** Cells were incubated with Antcin K (0.3, 1, 3 or 10 μM for 24 and cell viability was examined by the MTT assay. **(C–E)** Cells were incubated with Antcin K (10 μM) for 24 h and the expression of 40 inflammatory factors was quantified by a human inflammation antibody array. **(F)** Inhibitory effects of Antcin K on TNF-α, IL-1β, IL-6, IL-8 and protein secretion were evaluated by Western blot. **(G–I)** Cells were incubated with Antcin K (0.3, 1, 3 or 10 μM) for 24 h. TNF-α, IL-1β and IL-8 mRNA expression and protein secretion were evaluated by qPCR, Western blot, and ELISA. Results are expressed as the mean ± SD. *P < 0.05 compared with controls.

RASFs are critical in the initiation of proinflammatory cytokine secretion and their stimulation by proinflammatory cytokines influences cartilage and bone degradation in the RA microenvironment (28). In this study, we employed an immortalized RASF cell line (MH7A) that exhibits similar characteristics to human RASFs (29) and produces TNF-α (30–32), IL-1β (33–35) and IL-8 (35–38). According to MTT data, incubation of human RASFs for 24 h with Antcin K (0.3, 1, 3, or 10 μM) did not affect cell viability (**Figures 2A, B**), so we used all doses for investigating dose-dependent effects and only the highest concentration (10 μM) was used for the protein array and pathway activator analyses (**Figures 2C–E**). Findings from the human inflammation protein array showed that TNF-α, IL-1β and IL-8 expression was significantly inhibited in human RASFs after 24 h of incubation with Antcin K (10 μM) compared with vehicle (**Figures 2C–E**), which was also the case with our Western blot results (**Figure 2F**). In further experiments, IL-6 expression was similarly significantly inhibited after RASFs were incubated with Antcin K (10 μM) for 24 h (**Figures 2C–F**). Antcin K was associated with significant dose-dependent (0.3, 1, 3, or 10 μM) reductions in levels of TNF-α, IL-1β and IL-8 mRNA, as well as protein expression in RASFs (**Figures 2G–I**). Thus, Antcin K appears to dose-dependently inhibit the production of proinflammatory cytokines in human RASFs.

### The FAK/PI3K and AKT Signaling Cascades Are Implicated in Antcin K-Mediated Proinflammatory Cytokine Inhibition

**Figure 3:**
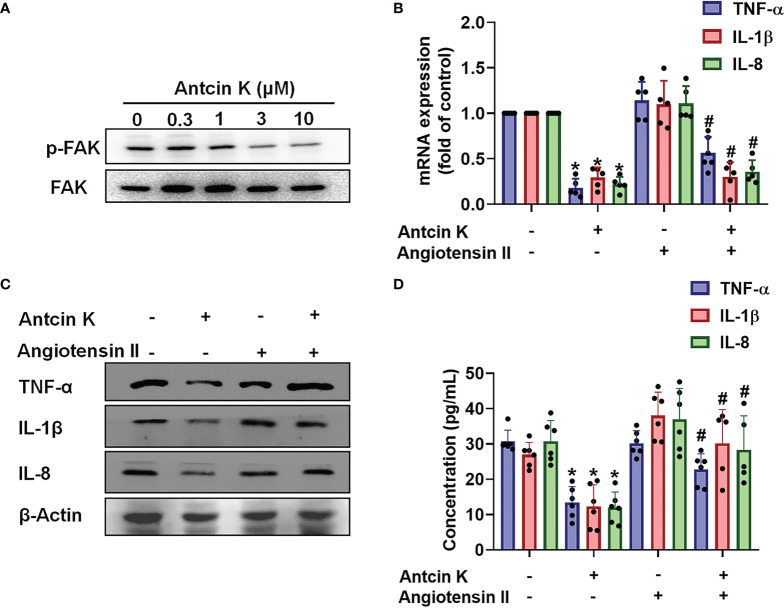
The FAK pathway is involved in Antcin K-induced inhibition of TNF-α, IL-1β and IL-8 production in RASFs. **(A)** Cells were treated with Antcin K (0.3, 1, 3 or 10 μM) for 24 h and FAK phosphorylation was examined by Western blot. **(B–D)** Cells were pretreated with the FAK activator Angiotensin II for 30 min, then treated with Antcin K (10 μM) for 24 h. TNF-α, IL-1β and IL-8 mRNA expression and protein secretion were examined by qPCR, Western blot and ELISA. Results are expressed as the mean ± SD. *P < 0.05 compared with controls; ^#^ *P* < 0.05 compared with the Antcin K-treated group.

**Figure 4:**
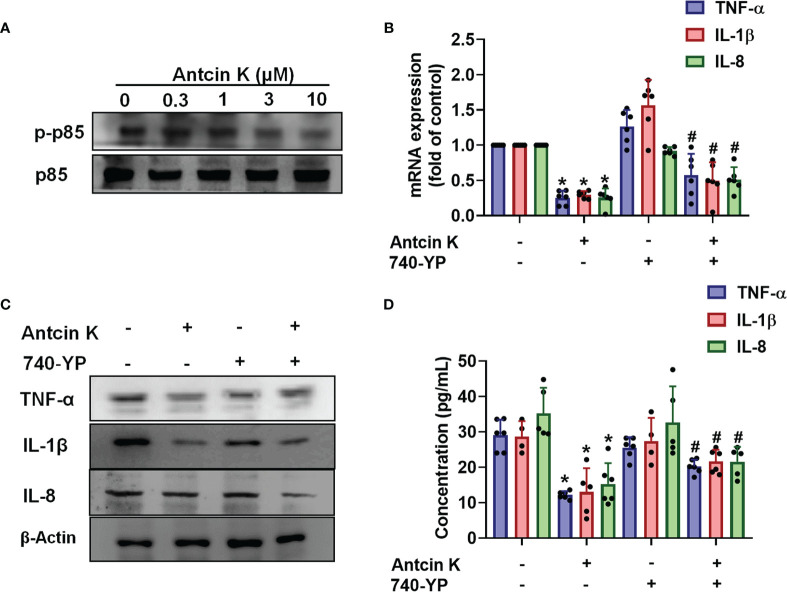
The PI3K pathway is involved in Antcin K-induced inhibition of TNF-α, IL-1β and IL-8 production in RASFs. **(A)** Cells were treated with Antcin K (0.3, 1, 3 or 10 μM) for 24 h and PI3K phosphorylation was examined by Western blot. **(B–D)** Cells were pretreated with PI3K activator 740-YP for 30 min, then treated with Antcin K (10 μM) for 24 h. TNF-α, IL-1β and IL-8 mRNA expression and protein secretion were examined by qPCR, Western blot and ELISA. Results are expressed as the mean ± SD. *P < 0.05 compared with controls; ^#^ *P* < 0.05 compared with the Antcin K-treated group.

**Figure 5:**
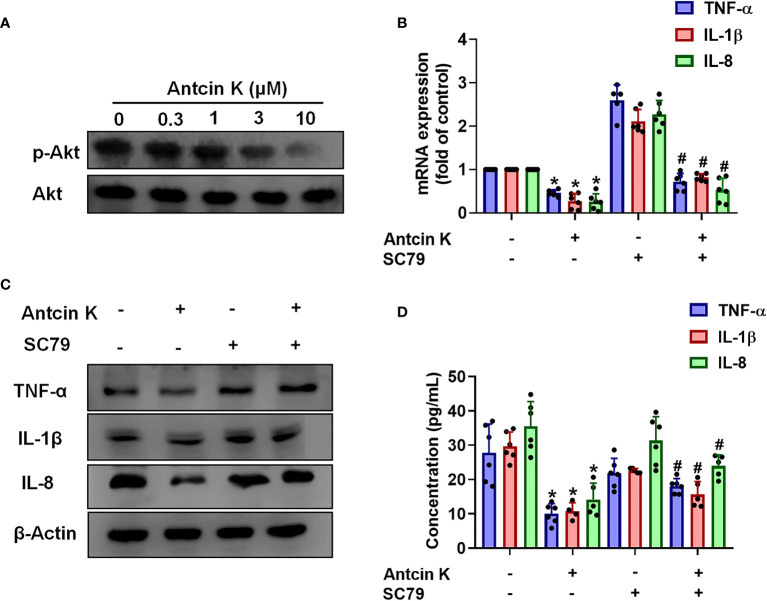
The AKT pathway is involved in Antcin K-induced inhibition of TNF-α, IL-1β and IL-8 production in synovial fibroblasts. **(A)** Cells were treated with Antcin K (0.3, 1, 3, or 10 μM) for 24 h and AKT phosphorylation was examined by Western blot. Cells were pretreated with an AKT activator (SC-79) for 30 min, then treated with Antcin K (10 μM) for 24 h. **(B–D)** TNF-α, IL-1β and IL-8 mRNA expression and protein secretion were examined by qPCR, Western blot and ELISA. Results are expressed as the mean ± SD. *P < 0.05 compared with controls; ^#^ *P* < 0.05 compared with the Antcin K-treated group.

The FAK/PI3K and AKT intracellular signaling pathways regulate cell growth and proliferation, and promote cytokine production (39). We examined whether FAK/PI3K and AKT signaling cascades interfere with the effects of Antcin K. Incubation of human RASFs with Antcin K (0.3, 1, 3, or 10 μM) for 24 h reduced the phosphorylation of FAK, PI3K and AKT (**Figures 3A**, **4A**, **5A**). Stimulating the RASFs with the FAK activator angiotensin, the PI3K activator 740-YP, and the AKT activator SC-79 (10 μM for each activator) significantly antagonized Antcin K-induced reductions of TNF-α, IL-1β and IL-8 production (**Figures 3**, **4**, **5B, C**). We then collected the cell culture medium from these treatments and we analyzed the levels of TNF-α, IL-1β and IL-8. Compared with untreated RASFs, levels of all three proinflammatory cytokines were significantly reduced in the RASFs treated with Antcin K (**Figures 3**–**5**). These findings suggest that Antcin K inhibits the production of these proinflammatory cytokines in RASFs *via* the FAK/PI3K and AKT signaling cascades.

### The NF-κB Pathway Is Implicated in Antcin K-Mediated Proinflammatory Cytokine Inhibition

**Figure 6:**
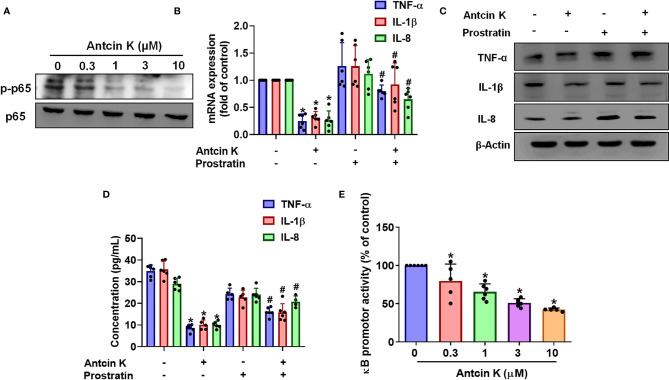
The NF-κB pathway is involved in Antcin K-induced inhibition of TNF-α, IL-1β and IL-8 production in RASFs. **(A)** Cells were treated with Antcin K (0.3, 1, 3 or 10 μM) for 24 h and NF-κB phosphorylation was examined by Western blot. Cells were pretreated with the NF-κB activator (prostratin) for 30 min, then treated with Antcin K (10 μM) for 24 h. **(B–D)** TNF-α, IL-1β and IL-8 mRNA expression and protein secretion were examined by qPCR, Western blot and ELISA. **(E)** Cells were transfected with the NF-κB luciferase plasmid, then incubated with Antcin K (0.3, 1, 3, or 10 μM) for 24 h and luciferase activity was determined. Results are expressed as the mean ± SD. *P < 0.05 compared with controls; ^#^ *P* < 0.05 compared with the Antcin K-treated group.

The NF-κB transcription factor is a critical player in inflammatory diseases (40) and is capable of activating several major proinflammatory cytokines including TNF-α, IL-1β and IL-8 (41, 42). We therefore examined whether Antcin K interferes with NF-κB phosphorylation. Treating RASFs with Antcin K (0.3, 1, 3, or 10 μM) for 24 h dose-dependently reduced NF-κB phosphorylation (**Figure 6A**). Prostratin rescued Antcin K-induced inhibition of TNF-α, IL-1β and IL-8 expression (**Figures 6B–D**). Transfecting the RASFs with the NF-κB luciferase plasmid after incubation with Antcin K (0.3, 1, 3, or 10 μM) for 24 h dose-dependently reduced NF-κB luciferase reporter activity (**Figure 6E**). Antcin K appears to inhibit proinflammatory cytokines by inhibiting the NF-κB signaling cascades.

### Antcin K Attenuates Signs and Symptoms of RA Disease in CIA Mice

**Figure 7:**
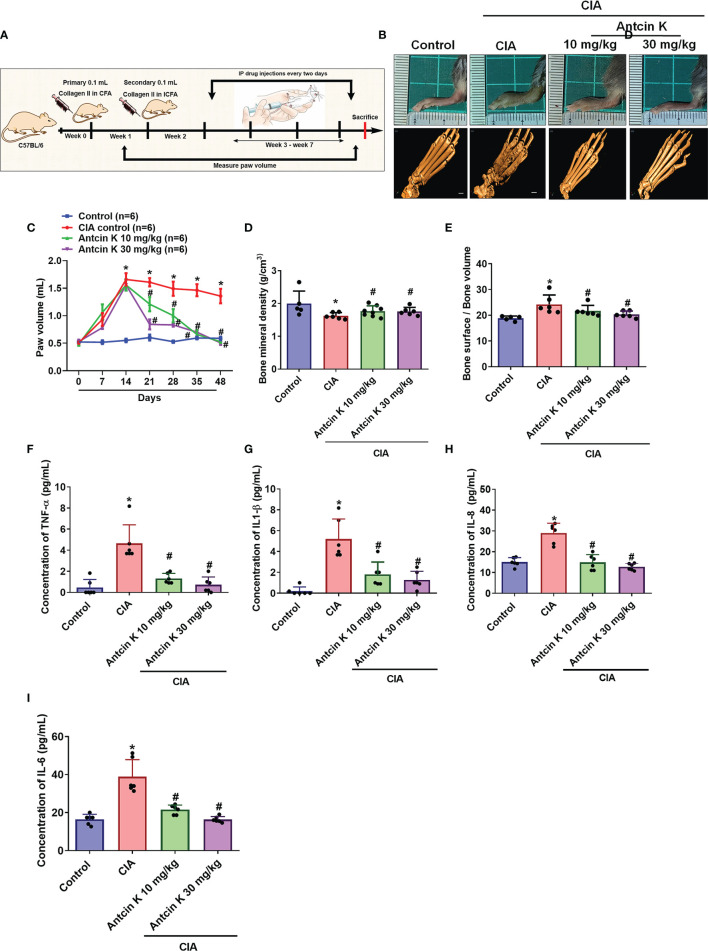
Antcin K ameliorated paw swelling and cartilage degradation in CIA mice. **(A)** Workflow of CIA induction and Antcin K injections. **(B, C)** Hind paw swelling was photographed and measured with a digital plethysmometer in healthy controls, untreated CIA mice, and in CIA mice administered Antcin K 10 mg/kg or 30 mg/kg for 48 weeks. Representative micro-CT images of the hind paws were recorded on Day 48. **(D, E)** Quantifications of bone volume, bone surface, and bone density. **(F–I)** Serum levels of TNF-α, IL-1β, IL-6 and IL-8 were analyzed by ELISA. Results are expressed as the mean ± SD. *P < 0.05 compared with controls; *^#^P* < 0.05 compared with the untreated CIA mice.

We next examined the anti-arthritic effects of Antcin K in CIA mice. Antcin K 10 mg/kg and 30 mg/kg significantly reduced the extents of paw swelling and bone degradation in CIA mice compared with controls (**Figures 7A–C**). Micro-CT imaging of the hind paws showed how Antcin K ameliorated bone erosion and enhanced bone mineral density (**Figures 7D, E**). Serum levels of TNF-α, IL-1β and IL-8 were significantly reduced in Antcin K-treated CIA mice (**Figures 7F–H**); notably, IL-6 levels were also significantly reduced in further experiments (**Figure 7I**).

**Figure 8:**
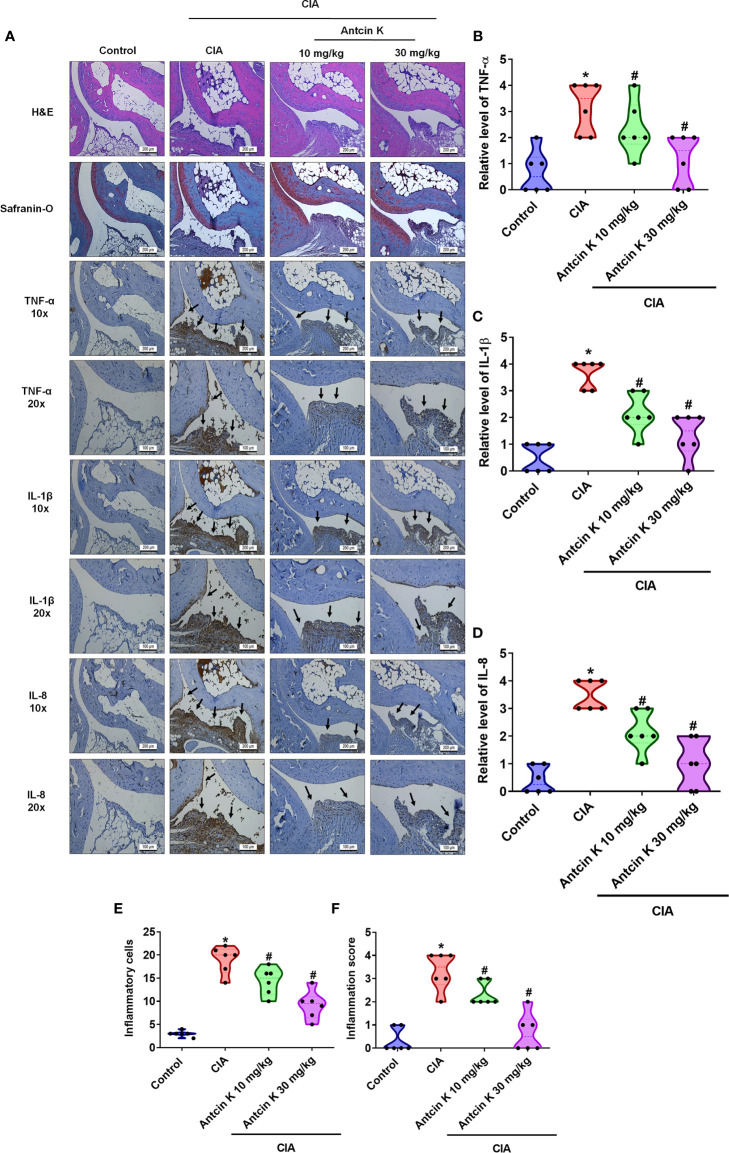
Effects of Antcin K quantified by histopathological changes in ankle joints from CIA mice. **(A)** Representative magnified images of histologic sections taken from ankle joints that were stained with H&E and Safranin-O, and IHC staining for TNF-α, IL-1β and IL-8 antibodies from healthy controls, untreated CIA mice, and CIA mice administered Antcin K 10 mg/kg or 30 mg/kg (N=6 per group). **(B–D)** Quantification of TNF-α, IL-1β and IL-8 expression by IHC score. **(E, F)** Quantification of inflammation scores and pathology findings in ankle joint synovium and adjacent tissue (details are provided in **Supplementary Table 1**). Results are expressed as the mean ± SD. *P < 0.05 compared with controls; *^#^P* < 0.05 compared with the untreated CIA mice.

IHC staining of synovium tissue revealed significantly higher TNF-α, IL-1β and IL-8 expression in CIA mice compared with controls; both concentrations of Antcin K (10 mg/kg and 30 mg/kg) significantly reduced the levels of all three cytokines (**Figures 8A–D**). Inflammatory cell infiltration into the synovial cell layers was significantly and dose-dependently reduced by Antcin K compared with untreated CIA mice (**Figure 8E** and **Supplementary Table 1**); synovium inflammation was also significantly and dose-dependently reduced by Antcin K compared with no treatment (**Figure 8F** and **Supplementary Table 1**). Thus, Antcin K appears to inhibit RA disease activity in CIA mice.

## Discussion

RA is associated with synovial inflammation and joint destruction (43). Onset of the disease is characterized by pannus formation and a hyperplastic synovium due to the deposition of synovial fibroblasts (10, 44). Compared with healthy fibroblasts, RASFs exhibit aggressive pathological characteristics in terms of structure and gene expression (45, 46). Moreover, activated RASFs exhibit a loss of contact inhibition (47) and accelerate the levels of adhesion molecules and proinflammatory cytokines including TNF-α, IL-1β and IL-8, causing synovial inflammation and cartilage damage (44).

Anti-TNF therapy has already proven effective in the treatment of RA (48), while IL-1β is also a key proinflammatory cytokine in inflammation-related diseases and cell injury, with disrupted IL-1β signaling accelerating the pathogenesis of inflammatory disease (49). As for IL-8, its abundant expression during inflammation promotes the formation of new blood vessels (50). Our study findings demonstrate high levels of TNF-α, IL-1β and IL-8 expression in human RA synovial tissue and plasma. As revealed by our CIA mouse model, the levels of these proinflammatory cytokines were closely associated with RA disease activity, with higher expression related to increasingly severe cartilage and bone degradation.

Several pharmacological inhibitors block these proinflammatory cytokines, including infliximab, etanercept, adalimumab and golimumab (51). However, loss of treatment responsiveness is common and side effects can develop that only subside after treatment discontinuation (52). Indigenous or traditional medicines involving herbal extracts are often considered to be ‘natural’ and causing fewer side effects than synthetic pharmacotherapies. The unique fungus *A. cinnamomea* has been used for hundreds of years in traditional medicines of Taiwan to treat liver diseases, food and drug intoxication, hypertension, cancers and inflammatory disorders, amongst other conditions (15, 53). Evidence has shown that extracts from *A. cinnamomea* have anti-inflammatory properties, such as the active compound ergostatrien-3β-ol (ST1), which significantly decreases mRNA levels of pro-inflammatory genes including *IL-6* and inducible nitric oxide synthase (*iNOS*) and levels of TNF-α, NF-κB and IL-6 protein expression, reducing inflammatory reactions after skin flap surgery (54). Moreover, when cultured in the solid state and in wood, *A. cinnamomea* protects the lung against hyperoxia-induced acute lung injury in mice by reducing hyperoxia-induced generation of reactive oxygen species (ROS) and suppresses IL-6, TNF-α, IL-1β, IL-8, IKKα/β and iNOS expression (55). Amongst the functional compounds derived from the fruiting bodies of *A. cinnamomea* (56), the findings of this suggest that the triterpenoid Antcin K mediates anti-inflammatory activity in RA disease with no apparent signs of cytotoxicity.

The FAK/PI3K and AKT signaling cascades initiate proinflammatory cytokine production (57, 58) and are important in inflammation to regulate multicellular functions at the site of synovial proliferation and promote cytokine production from RASFs (59, 60). Antcin K treatment inhibits the migration and invasion of human hepatoma cells by suppressing the phosphorylation of FAK, PI3K and AKT signaling (61). Our study demonstrates that Antcin K downregulates FAK/PI3K and AKT phosphorylation. When we subsequently used specific activators to upregulate TNF-α, IL-1β and IL-8 expression, Antcin K treatment restored the levels to normal. These findings suggest that FAK/PI3K and AKT intracellular signaling cascades are pivotal in Antcin K-mediated proinflammatory cytokine production in RASFs.

NF-κB is a transcription factor that is not only vital in inflammatory responses, but also is a key molecule in the link between chronic inflammation and cancer (41, 62). We therefore examined whether Antcin K interferes with the NF-κB pathway. Interestingly, we found that 24 h of Antcin K treatment inhibited NF-κB phosphorylation and this activity was rescued by the NF-κB activator prostratin. Moreover, when we transfected RASFs with the NF-κB luciferase plasmid, we found that Antcin K dose-dependently inhibited NF-κB luciferase activity. These findings suggest that Antcin K has potential for blocking proinflammatory cytokine production *via* the inhibition of the FAK/PI3K, AKT and NF-κB signaling cascades.

Structural cartilage and bone degradation are the hallmarks of RA, with circulating RASFs promoting this damage to unaffected joints (63), contributing to the production of cytokines and proteolytic enzymes that degrade the extracellular matrix (45). Our experimental findings reveal that CIA mice administered intraperitoneal injections of Antcin K (10 mg/kg or 30 mg/kg) exhibit reductions in paw swelling, bone erosion and cartilage bone degradation, as well as lower serum levels of proinflammatory cytokines TNF-α, IL-1β, IL-8 and IL-6 compared with normal controls.

**Figure 9:**
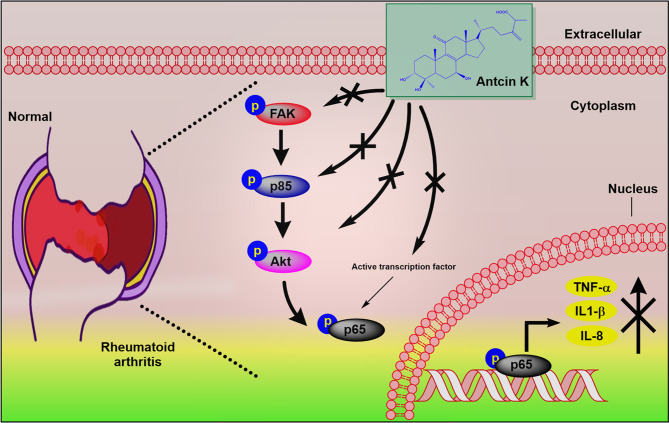
Schematic diagram summarizes the mechanism whereby Antcin K inhibits proinflammatory cytokine production. Antcin K inhibits TNF-α, IL-1β and IL-8 production in human RA synovial fibroblasts and in serum and synovial tissue from CIA mice. These anti-inflammatory effects occur through the FAK/PI3K, AKT, and NF-κB signaling cascades.

In conclusion, our observations indicate that the inhibition of TNF-α, IL-1β and IL-8 production in RASFs and in serum and synovial tissue from CIA mice attenuates signs and symptoms of RA (**Figure 9**) and that Antcin K significantly inhibits these proinflammatory cytokines *via* the FAK/PI3K, AKT and NF-κB signaling cascades. Derivatives of *A. cinnamomea* have exhibited varying pharmacological activity in experimental models of tumors, inflammation, immunomodulation, hepatotoxicity, diabetes and hyperlipidemia (53). In one study, triterpenoids isolated from *A. cinnamomea* mycelia were protective against acute alcohol-induced liver injury in mice (64) and in another study, *A. cinnamomea* polysaccharides inhibited vascular endothelial growth factor receptor (VEGFR) signaling in bovine aortic endothelial cells (65). This study has shown for the first time that the *A. cinnamomea* extract Antcin K inhibits RA disease activity in human RASFs and in a mouse model of RA. These findings offer new opportunities for RA disease management.

## Institutional Review Board Statement

The animal study and protocols were approved by the Institutional Animal Care and Use Committee of China Medical University (IACUC Approval No: 2016-295).

## Data Availability Statement

The datasets presented in this study can be found in online repositories. The names of the repository/repositories and accession number(s) can be found in the article/**Supplementary Material**.

## Ethics Statement

The study protocol was approved by the Institutional Review Board of China Medical University Hospital (CMUH108-REC3-039). Written informed consent to participate in this study was provided by the participants’ legal guardian/next of kin. The animal study and protocols were approved by the Institutional Animal Care and Use Committee of China Medical University (IACUC Approval No: 2016-295). Written informed consent was obtained from the owners for the participation of their animals in this study.

## Author Contributions

DA and C-HTa conceived and designed the project. Methodology was performed by DA and S-CL. Experiments and data analysis were performed by DA and Y-YL. Histological analysis and interpreted the results by I-PC. Reagents, materials and analytical tools were supplied by C-HTa and Y-HK. Funding was acquired by C-HTa. The paper was written by DA and revised by C-CH, C-HTs, C-YK, and C-HTa. All authors contributed to the article and approved the submitted version.

## Funding

This work was supported by grants from the Ministry of Science and Technology in Taiwan (MOST 110-2320-B-039-022-MY3; MOST 110-2314-B-039-008; MOST 110-2314-B-039-012), and China Medical University under the Higher Education Sprout Project, Ministry of Education, Taiwan (CMRC-CHM-2-2) and China Medical University Hospital (DMR-111-165; DMR-111-117; DMR-111-028).

## Conflict of Interest

The authors declare that the research was conducted in the absence of any commercial or financial relationships that could be construed as a potential conflict of interest.

## Publisher’s Note

All claims expressed in this article are solely those of the authors and do not necessarily represent those of their affiliated organizations, or those of the publisher, the editors and the reviewers. Any product that may be evaluated in this article, or claim that may be made by its manufacturer, is not guaranteed or endorsed by the publisher.

## Abbreviations

*A. cinnamomea, Antrodia cinnamomea;* RA, rheumatoid arthritis; RASF, rheumatoid arthritis synovial fibroblast; FAK, focal adhesion kinase; PI3K, phosphoinositide 3-kinase; AKT, protein kinase B; NF-κB, nuclear factor-κB; CIA, collagen-induced arthritis.

## References

[1] Coutant F Miossec P. Evolving Concepts of the Pathogenesis of Rheumatoid Arthritis With Focus on the Early and Late Stages. Curr Opin Rheumatol (2020) 32(1):57–63. doi: 10.1097/BOR.0000000000000664 31644463

[2] Deane KD Holers VM. Rheumatoid Arthritis Pathogenesis, Prediction, and Prevention: An Emerging Paradigm Shift. Arthritis Rheumatol (2021) 73(2):181–93. doi: 10.1002/art.41417 PMC7772259 32602263

[3] Aghakhani S Zerrouk N Niarakis A. Metabolic Reprogramming of Fibroblasts as Therapeutic Target in Rheumatoid Arthritis and Cancer: Deciphering Key Mechanisms Using Computational Systems Biology Approaches. Cancers (Basel) (2020) 13(1):35. doi: 10.3390/cancers13010035 PMC7795338 33374292

[4] Falconer J Murphy AN Young SP Clark AR Tiziani S Guma M. Review: Synovial Cell Metabolism and Chronic Inflammation in Rheumatoid Arthritis. Arthritis Rheumatol (2018) 70(7):984–99. doi: 10.1002/art.40504 PMC6019623 29579371

[5] Yoshitomi H. Regulation of Immune Responses and Chronic Inflammation by Fibroblast-Like Synoviocytes. Front Immunol (2019) 10:1395. doi: 10.3389/fimmu.2019.01395 31275325 PMC6593115

[6] Paglia MD Silva MT Lopes LC Barberato S Mazzei LG Abe FC. Use of Corticoids and Non-Steroidal Anti-Inflammatories in the Treatment of Rheumatoid Arthritis: Systematic Review and Network Meta-Analysis. PloS One (2021) 16(4):e0248866. doi: 10.1371/journal.pone.0248866 33826610 PMC8026036

[7] Burmester GR Pope JE. Novel Treatment Strategies in Rheumatoid Arthritis. Lancet (2017) 389(10086):2338–48. doi: 10.1016/S0140-6736(17)31491-5 28612748

[8] Luis M Freitas J Costa F Buttgereit F Boers M Da Silva JAP. An Updated Review of Glucocorticoid-Related Adverse Events in Patients With Rheumatoid Arthritis. Expert Opin Drug Saf (2019) 18(7):581–90. doi: 10.1080/14740338.2019.1615052 31056959

[9] Rubbert-Roth A Szabo MZ Kedves M Nagy G Atzeni F Sarzi-Puttini P. Failure of Anti-TNF Treatment in Patients With Rheumatoid Arthritis: The Pros and Cons of the Early Use of Alternative Biological Agents. Autoimmun Rev (2019) 18(12):102398. doi: 10.1016/j.autrev.2019.102398 31639514

[10] Guo Q Wang YX Xu D Nossent J Pavlos NJ Xu JK. Rheumatoid Arthritis: Pathological Mechanisms and Modern Pharmacologic Therapies. Bone Res (2018) 6(1):1–14. doi: 10.1038/s41413-018-0016-9 29736302 PMC5920070

[11] Dong Y Chen HW Gao JL Liu YM Li J Wang J. Bioactive Ingredients in Chinese Herbal Medicines That Target Non-Coding RNAs: Promising New Choices for Disease Treatment. Front Pharmacol (2019) 10. doi: 10.3389/fphar.2019.00515 PMC6537929 31178721

[12] Li RF Hou YL Huang JC Pan WQ Ma QH Shi YX. Lianhuaqingwen Exerts Anti-Viral and Anti-Inflammatory Activity Against Novel Coronavirus (SARS-CoV-2). Pharmacol Res (2020) 156:104761. doi: 10.1016/j.phrs.2020.104761 32205232 PMC7102548

[13] Ganesan N Baskaran R Velmurugan BK Thanh NC. Antrodia Cinnamomea-An Updated Minireview of Its Bioactive Components and Biological Activity. J Food Biochem (2019) 43(8):e12936. doi: 10.1111/jfbc.12936 31368557

[14] Chen YY Chou PY Chien YC Wu CH Wu TS Sheu MJ. Ethanol Extracts of Fruiting Bodies of Antrodia Cinnamomea Exhibit Anti-Migration Action in Human Adenocarcinoma CL1-0 Cells Through the MAPK and PI3K/AKT Signaling Pathways. Phytomedicine (2012) 19(8-9):768–78. doi: 10.1016/j.phymed.2012.02.016 22464013

[15] Huang TT Wu SP Chong KY Ojcius DM Ko YF Wu YH. The Medicinal Fungus Antrodia Cinnamomea Suppresses Inflammation by Inhibiting the NLRP3 Inflammasome. J Ethnopharmacol (2014) 155(1):154–64. doi: 10.1016/j.jep.2014.04.053 24858059

[16] Tang CH Hsu CJ Fong YC. The CCL5/CCR5 Axis Promotes Interleukin-6 Production in Human Synovial Fibroblasts. Arthritis Rheum-Us (2010) 62(12):3615–24. doi: 10.1002/art.27755 20862675

[17] Lien MY Tsai HC Chang AC Tsai MH Hua CH Wang SW. Chemokine CCL4 Induces Vascular Endothelial Growth Factor C Expression and Lymphangiogenesis by miR-195-3p in Oral Squamous Cell Carcinoma. Front Immunol (2018) 9:412. doi: 10.3389/fimmu.2018.00412 29599774 PMC5863517

[18] Lee H-P Chen P-C Wang S-W Fong Y-C Tsai C-H Tsai F-J. Plumbagin Suppresses Endothelial Progenitor Cell-Related Angiogenesis *In Vitro* and *In Vivo* . J Funct Foods (2019) 52:537–44. doi: 10.1016/j.jff.2018.11.040

[19] Liu S-C Tsai C-H Wu T-Y Tsai C-H Tsai F-J Chung J-G. Soya-Cerebroside Reduces IL-1β-Induced MMP-1 Production in Chondrocytes and Inhibits Cartilage Degradation: Implications for the Treatment of Osteoarthritis. Food Agr Immunol (2019) 30(1):620–32. doi: 10.1080/09540105.2019.1611745

[20] Lee H-P Wang S-W Wu Y-C Tsai C-H Tsai F-J Chung J-G. Glucocerebroside Reduces Endothelial Progenitor Cell-Induced Angiogenesis. Food Agr Immunol (2019) 30(1):1033–45. doi: 10.1080/09540105.2019.1660623

[21] Lee H-P Liu S-C Wang Y-H Chen B-C Chen H-T Li T-M. Cordycerebroside A Suppresses VCAM-Dependent Monocyte Adhesion in Osteoarthritis Synovial Fibroblasts by Inhibiting MEK/ERK/AP-1 Signaling. J Funct Foods (2021) 86:104712. doi: 10.1016/j.jff.2021.104712

[22] Su C-H Lin C-Y Tsai C-H Lee H-P Lo L-C Huang W-C. Betulin Suppresses TNF-α and IL-1β Production in Osteoarthritis Synovial Fibroblasts by Inhibiting the MEK/ERK/NF-κb Pathway. J Funct Foods (2021) 86:104729. doi: 10.1016/j.jff.2021.104729

[23] Huang CC Chiou CH Liu SC Hu SL Su CM Tsai CH. Melatonin Attenuates TNF-α and IL-1β Expression in Synovial Fibroblasts and Diminishes Cartilage Degradation: Implications for the Treatment of Rheumatoid Arthritis. J Pineal Res (2019) 66(3):e12560. doi: 10.1111/jpi.12560 30648758

[24] Hu SL Chang AC Huang CC Tsai CH Lin CC Tang CH. Myostatin Promotes Interleukin-1β Expression in Rheumatoid Arthritis Synovial Fibroblasts Through Inhibition of miR-21-5p. Front Immunol (2017) 8:1747. doi: 10.3389/fimmu.2017.01747 29276516 PMC5727021

[25] Fechtner S Singh AK Srivastava I Szlenk CT Muench TR Natesan S. Cannabinoid Receptor 2 Agonist JWH-015 Inhibits Interleukin-1 Beta-Induced Inflammation in Rheumatoid Arthritis Synovial Fibroblasts and in Adjuvant Induced Arthritis Rat *via* Glucocorticoid Receptor. Front Immunol (2019) 10. doi: 10.3389/fimmu.2019.01027 PMC6519139 31139184

[26] Singh AK Haque M O’Sullivan K Chourasia M Ouseph MM Ahmed S. Suppression of Monosodium Urate Crystal-Induced Inflammation by Inhibiting TGF-Beta-Activated Kinase 1-Dependent Signaling: Role of the Ubiquitin Proteasome System. Cell Mol Immunol (2021) 18(1):162–70. doi: 10.1038/s41423-019-0284-3 PMC7853128 31511642

[27] Epsley S Tadros S Farid A Kargilis D Mehta S Rajapakse CS. The Effect of Inflammation on Bone. Front Physiol (2020) 11:511799. doi: 10.3389/fphys.2020.511799 33584321 PMC7874051

[28] Ospelt C. Synovial Fibroblasts in 2017. RMD Open (2017) 3(2):e000471. doi: 10.1136/rmdopen-2017-000471 29081987 PMC5652455

[29] Miyazawa K Mori A Okudaira H. Establishment and Characterization of a Novel Human Rheumatoid Fibroblast-Like Synoviocyte Line, MH7A, Immortalized With SV40 T Antigen. J Biochem (1998) 124(6):1153–62. doi: 10.1093/oxfordjournals.jbchem.a022233 9832620

[30] Wang J Zhao Q. Kaempferitrin Inhibits Proliferation, Induces Apoptosis, and Ameliorates Inflammation in Human Rheumatoid Arthritis Fibroblast-Like Synoviocytes. Phytother Res (2019) 33(6):1726–35. doi: 10.1002/ptr.6364 31155798

[31] Someya A Ikegami T Sakamoto K Nagaoka I. Glucosamine Downregulates the IL-1β-Induced Expression of Proinflammatory Cytokine Genes in Human Synovial MH7A Cells by O-GlcNAc Modification-Dependent and-Independent Mechanisms. PloS One (2016) 11(10):e0165158. doi: 10.1371/journal.pone.0165158 27776166 PMC5077170

[32] Meng D Li J Li H Wang K. Salvianolic Acid B Remits LPS-Induced Injury by Up-Regulating miR-142-3p in MH7A Cells. BioMed Pharmacother (2019) 115:108876. doi: 10.1016/j.biopha.2019.108876 31022596

[33] Li J Li J Yue Y Hu Y Cheng W Liu R. Genistein Suppresses Tumor Necrosis Factor Alpha-Induced Inflammation *via* Modulating Reactive Oxygen Species/Akt/Nuclear Factor kappaB and Adenosine Monophosphate-Activated Protein Kinase Signal Pathways in Human Synoviocyte MH7A Cells. Drug Des Devel Ther (2014) 8:315–23. doi: 10.2147/DDDT.S52354 PMC3962316 24669186

[34] Liu Z Song L Wang Y Xu P Guo X Yang J. A Novel Fusion Protein Attenuates Collagen-Induced Arthritis by Targeting Interleukin 17A and Tumor Necrosis Factor Alpha. Int J Pharm (2018) 547(1-2):72–82. doi: 10.1016/j.ijpharm.2018.05.058 29803792

[35] Kong L Wang L Zhao Q Di G Wu H. Rhodojaponin II Inhibits TNF-Alpha-Induced Inflammatory Cytokine Secretion in MH7A Human Rheumatoid Arthritis Fibroblast-Like Synoviocytes. J Biochem Mol Toxicol (2020) 34(10):e22551. doi: 10.1002/jbt.22551 32613688

[36] Li L Li Y Shi D Liu H Wang B Sun Y. Xuebijing Inhibit Inflammation, Oxidative Stress and Promote Apoptosis in Human Synovial Cells *via* Inhibition of MEK1/2 and NF-ĸB Pathway. Israel Journal of Plant Sciences (2021) 1(aop):1–9. doi: 10.1163/22238980-bja10032

[37] Meng C Xia Q Wu H Huang H Liu H Li Y. Photobiomodulation With 630-Nm LED Radiation Inhibits the Proliferation of Human Synoviocyte MH7A Cells Possibly *via* TRPV4/PI3K/AKT/mTOR Signaling Pathway. Lasers Med Sci (2020) 35(9):1927–36. doi: 10.1007/s10103-020-02977-5 32162133

[38] Zhang Q Peng W Wei S Wei D Li R Liu J. Guizhi-Shaoyao-Zhimu Decoction Possesses Anti-Arthritic Effects on Type II Collagen-Induced Arthritis in Rats *via* Suppression of Inflammatory Reactions, Inhibition of Invasion & Migration and Induction of Apoptosis in Synovial Fibroblasts. BioMed Pharmacother (2019) 118:109367. doi: 10.1016/j.biopha.2019.109367 31545276

[39] Sun X Wang X Chen T Li T Cao K Lu A. Myelin Activates FAK/Akt/NF-kappaB Pathways and Provokes CR3-Dependent Inflammatory Response in Murine System. PloS One (2010) 5(2):e9380. doi: 10.1371/journal.pone.0009380 20186338 PMC2826415

[40] Park MH Hong JT. Roles of NF-κb in Cancer and Inflammatory Diseases and Their Therapeutic Approaches. Cells (2016) 5(2):15. doi: 10.3390/cells5020015 PMC4931664 27043634

[41] Liu T Zhang L Joo D Sun SC. NF-kappaB Signaling in Inflammation. Signal Transduct Target Ther (2017) 2(1):1–9. doi: 10.1038/sigtrans.2017.23 PMC5661633 29158945

[42] Simmonds RE Foxwell BM. Signalling, Inflammation and Arthritis: NF-kappaB and Its Relevance to Arthritis and Inflammation. Rheumatol (Oxford) (2008) 47(5):584–90. doi: 10.1093/rheumatology/kem298 18234712

[43] Edilova MI Akram A Abdul-Sater AA. Innate Immunity Drives Pathogenesis of Rheumatoid Arthritis. BioMed J (2021) 44(2):172–82. doi: 10.1016/j.bj.2020.06.010 PMC8178572 32798211

[44] Juarez M Filer A Buckley CD. Fibroblasts as Therapeutic Targets in Rheumatoid Arthritis and Cancer. Swiss Med Wkly (2012) 142:w13529. doi: 10.4414/smw.2012.13529 22367980

[45] Bartok B Firestein GS. Fibroblast-Like Synoviocytes: Key Effector Cells in Rheumatoid Arthritis. Immunol Rev (2010) 233(1):233–55. doi: 10.1111/j.0105-2896.2009.00859.x PMC2913689 20193003

[46] Lefevre S Meier FM Neumann E Muller-Ladner U. Role of Synovial Fibroblasts in Rheumatoid Arthritis. Curr Pharm Des (2015) 21(2):130–41. doi: 10.2174/1381612820666140825122036 25163744

[47] Seemayer CA Kuchen S Kuenzler P Rihoskova V Rethage J Aicher WK. Cartilage Destruction Mediated by Synovial Fibroblasts Does Not Depend on Proliferation in Rheumatoid Arthritis. Am J Pathol (2003) 162(5):1549–57. doi: 10.1016/S0002-9440(10)64289-7 PMC1851181 12707039

[48] Feldmann M Maini RN Woody JN Holgate ST Winter G Rowland M. Trials of Anti-Tumour Necrosis Factor Therapy for COVID-19 Are Urgently Needed. Lancet (2020) 395(10234):1407–9. doi: 10.1016/S0140-6736(20)30858-8 PMC7158940 32278362

[49] Kaneko N Kurata M Yamamoto T Morikawa S Masumoto J. The Role of Interleukin-1 in General Pathology. Inflammation Regener (2019) 39:12. doi: 10.1186/s41232-019-0101-5 PMC6551897 31182982

[50] Azizi G Boghozian R Mirshafiey A. The Potential Role of Angiogenic Factors in Rheumatoid Arthritis. Int J Rheum Dis (2014) 17(4):369–83. doi: 10.1111/1756-185X.12280 24467605

[51] Koenders MI van den Berg WB. Novel Therapeutic Targets in Rheumatoid Arthritis. Trends Pharmacol Sci (2015) 36(4):189–95. doi: 10.1016/j.tips.2015.02.001 25732812

[52] Smolen JS Aletaha D McInnes IB. Rheumatoid Arthritis. Lancet (2016) 388(10055):2023–38. doi: 10.1016/S0140-6736(16)30173-8 27156434

[53] Zhang BB Guan YY Hu PF Chen L Xu GR Liu LM. Production of Bioactive Metabolites by Submerged Fermentation of the Medicinal Mushroom Antrodia Cinnamomea: Recent Advances and Future Development. Crit Rev Biotechnol (2019) 39(4):541–54. doi: 10.1080/07388551.2019.1577798 30810393

[54] Tsai TC Tung YT Kuo YH Liao JW Tsai HC Chong KY. Anti-Inflammatory Effects of Antrodia Camphorata, a Herbal Medicine, in a Mouse Skin Ischemia Model. J Ethnopharmacol (2015) 159:113–21. doi: 10.1016/j.jep.2014.11.015 25449461

[55] Tung YT Tsai TC Kuo YH Yen CC Sun JY Chang WH. Comparison of Solid-State-Cultured and Wood-Cultured Antrodia Camphorata in Anti-Inflammatory Effects Using NF-κb/Luciferase Inducible Transgenic Mice. Phytomedicine (2014) 21(12):1708–16. doi: 10.1016/j.phymed.2014.08.011 25442281

[56] Lu MC El-Shazly M Wu TY Du YC Chang TT Chen CF. Recent Research and Development of Antrodia Cinnamomea. Pharmacol Therapeut (2013) 139(2):124–56. doi: 10.1016/j.pharmthera.2013.04.001 23563277

[57] Brister D Aulakah G Zhang H Patel K. Blocking Focal Adhesion Kinase (FAK) Protects Against Damage in a Novel Model of Lymphocyte Dominant Lung Injury. Eur Respir J (2020) 56. doi: 10.1183/13993003.congress-2020.4469

[58] Xu P Wang J Yang ZW Lou XL Chen C. Regulatory Roles of the PI3K/Akt Signaling Pathway in Rats With Severe Acute Pancreatitis. PloS One (2013) 8(11):e81767. doi: 10.1371/journal.pone.0081767 24312352 PMC3842964

[59] Xie L Xie H Chen C Tao Z Zhang C Cai L. Inhibiting the PI3K/AKT/NF-kappaB Signal Pathway With Nobiletin for Attenuating the Development of Osteoarthritis: *In Vitro* and *In Vivo* Studies. Food Funct (2019) 10(4):2161–75. doi: 10.1039/C8FO01786G 30938722

[60] Liu Y Pan YF Xue YQ Fang LK Guo XH Guo X. uPAR Promotes Tumor-Like Biologic Behaviors of Fibroblast-Like Synoviocytes Through PI3K/Akt Signaling Pathway in Patients With Rheumatoid Arthritis. Cell Mol Immunol (2018) 15(2):171–81. doi: 10.1038/cmi.2016.60 PMC5811678 28090093

[61] Huang YL Chu YL Ho CT Chung JG Lai CI Su YC. An Active Triterpenoid From the Fruiting Bodies of Basswood-Cultivated Antrodia Cinnamomea, Inhibits Metastasis *via* Suppression of Integrin-Mediated Adhesion, Migration, and Invasion in Human Hepatoma Cells. J Agric Food Chem (2015) 63(18):4561–9. doi: 10.1021/jf5059304 25911944

[62] Taniguchi K Karin M. NF-Kappab, Inflammation, Immunity and Cancer: Coming of Age. Nat Rev Immunol (2018) 18(5):309–24. doi: 10.1038/nri.2017.142 29379212

[63] Hillen J Geyer C Heitzmann M Beckmann D Krause A Winkler I. Structural Cartilage Damage Attracts Circulating Rheumatoid Arthritis Synovial Fibroblasts Into Affected Joints. Arthritis Res Ther (2017) 19(1):40. doi: 10.1186/s13075-017-1245-9 28245866 PMC5331726

[64] Liu Y Wang Z Kong F Teng L Zheng X Liu X. Triterpenoids Extracted From Antrodia Cinnamomea Mycelia Attenuate Acute Alcohol-Induced Liver Injury in C57BL/6 Mice *via* Suppression Inflammatory Response. Front Microbiol (2020) 11:1113. doi: 10.3389/fmicb.2020.01113 32719658 PMC7350611

[65] Han HF Nakamura N Zuo F Hirakawa A Yokozawa T Hattori M. Protective Effects of a Neutral Polysaccharide Isolated From the Mycelium of Antrodia Cinnamomea on Propionibacterium Acnes and Lipopolysaccharide Induced Hepatic Injury in Mice. Chem Pharm Bull (Tokyo) (2006) 54(4):496–500. doi: 10.1248/cpb.54.496 16595952

