# Supplementary material for: Antcin K Inhibits TNF-α, IL-1β and IL-8 Expression in Synovial Fibroblasts and Ameliorates Cartilage Degradation: Implications for the Treatment of Rheumatoid Arthritis
Source: Front Immunol. 2021 Dec 15;12:790925. doi: 10.3389/fimmu.2021.790925 (PMC8714747; doi:10.3389/fimmu.2021.790925)
Supplement: Supplementary file 1 [file Table_1.docx]

**Supplementary data**

**Supplementary Table 1. Microscopic evaluation of inflammation scores and detailed analysis of pathology findings in ankle joint synovium and adjacent tissue from healthy control mice and CIA mice (untreated or treated with Antcin K) in slides stained with H&E.** All of the slides were independently evaluated by two pathologists blinded to treatment.
